# Validation and Application of a Low-Cost Sorting Device for Fumonisin Reduction in Maize

**DOI:** 10.3390/toxins13090652

**Published:** 2021-09-14

**Authors:** William Stafstrom, Julie Wushensky, John Fuchs, Wenwei Xu, Nnenna Ezera, Rebecca J. Nelson

**Affiliations:** 1 School of Integrative Plant Science, Cornell University, Ithaca, NY 14853, USA; wcs98@cornell.edu; 2 College of Engineering, Cornell University, Ithaca, NY 14853, USA; julie.wushensky@beyondmeat.com; 3 The Widget Factory, Ithaca, NY 14850, USA; jfuchs1@twcny.rr.com; 4 Agricultural Research and Extension Center, Texas A&M AgriLife Research, Lubbock, TX 79403, USA; wxu@ag.tamu.edu; 5 Cornell University, Ithaca, NY 14853, USA; nme26@cornell.edu

**Keywords:** mycotoxins, maize, fumonisin, sorting, food safety, mechanization

## Abstract

Fumonisin mycotoxins are a persistent challenge to human and livestock health in tropical and sub-tropical maize cropping systems, and more efficient methods are needed to reduce their presence in food systems. We constructed a novel, low-cost device for sorting grain, the “DropSort”, and tested its effectiveness on both plastic kernel models and fumonisin-contaminated maize. Sorting plastic kernels of known size and shape enabled us to optimize the sorting performance of the DropSort. The device sorted maize into three distinct fractions as measured by bulk density and 100-kernel weight. The level of fumonisin was lower in the heaviest fractions of maize compared to the unsorted samples. Based on correlations among fumonisin and bulk characteristics of each fraction, we found that light fraction 100-kernel weight could be an inexpensive proxy for unsorted fumonisin concentration. Single kernel analysis revealed significant relationships among kernel fumonisin content and physical characteristics that could prove useful for future sorting efforts. The availability of a low-cost device (materials~USD 300) that can be used to reduce fumonisin in maize could improve food safety in resource-limited contexts in which fumonisin contamination remains a pressing challenge.

## 1. Introduction

Fungal infection and mycotoxin contamination of crops is a global food safety hazard. The fumonisins, a class of toxic secondary fungal metabolites (hereafter collectively referred to as “fumonisin”) produced primarily by *Fusarium* spp., are some of the most ubiquitous mycotoxins in maize. Fumonisin presents challenges across a range of agricultural systems and is most prevalent in tropical and sub-tropical food systems where the fungal pathogen *F. verticillioides* frequently causes Fusarium ear rot (FER) on maize [1,2,3]. In humans, fumonisin exposure is associated with neural tube defects, esophageal cancer, and childhood stunting in humans [4,5,6,7,8]. For human consumption, the Codex Alimentarius Commission establishes a maximum level of 4 mg/kg (ppm) of fumonisin in raw maize grain, and the same concentration is the guidance level recommended by the United States Food and Drug Administration (FDA) for whole dry-milled maize [9,10].

In many low- and middle-income countries, mycotoxin regulations that exist are infrequently enforced, and there are significant challenges related to food safety and food security [11,12,13,14]. In areas like sub-Saharan Africa where maize is a major staple and environmental conditions are conducive to *Fusarium* spp. Growth, high fumonisin exposure is common [15,16,17]. Moreover, fumonisins can also co-contaminate maize with other mycotoxins such as the even more toxic aflatoxins [18,19]. Strategies to mitigate fumonisins have focused on prevention or reduction at multiple points along the food value chain, such as breeding and releasing resistant varieties, using good agronomic practices, biological controls, pesticide applications, optimizing storage conditions, improving sorting and cleaning operations, post-harvest enzymatic detoxification, and a wide variety of processing and cooking techniques [20,21,22]. Hazard analysis and critical control point (HACCP) approaches for mycotoxin mitigation have been developed to integrate such strategies, and sorting is frequently included as an essential step [23].

In subsistence and semi-subsistence agricultural systems, significant resource constraints limit the fumonisin mitigation options that can be employed. In a South African farming community, low-input hand-sorting and washing procedures for fumonisin-contaminated maize significantly reduced fumonisin exposure [24]. In various other low-resource contexts, manually removing visibly diseased or damaged maize kernels alone or in combination with other processes has proven to reduce fumonisin levels, although not always to below regulatory limits [25,26,27,28,29,30]. Although hand sorting has been proven effective for reducing fumonisin levels in maize, it is a time-consuming process. Furthermore, legitimate food security concerns limit the implementation of sorting methods that remove large amounts of potentially toxic grain [31,32].

Efforts to mechanize aspects of small-scale agriculture are crucial to development in rural communities. Simple machinery can provide opportunities for efficiency gains, job creation, improved markets, and quality products [33]. Mechanization of tasks typically ascribed to women can reduce the burdens on women’s time, which can have important implications on child wellbeing and nutrition [34]. In high-income countries, industrial-scale methods of cleaning and sorting mycotoxin-contaminated maize rely on advanced technologies that rapidly detect and sort out putatively toxic kernels. The bases of these mycotoxin-sorting operations are multi-step processes that select against kernels with features associated with mycotoxin contamination. For instance, the Bühler Group has developed various automated sorters that reject maize kernels based on size, shape, density, and/or optical features (Bühler Group, Uzwil, Switzerland). Promisingly, these machines can significantly reduce maize aflatoxin levels [35]. Moreover, existing post-harvest technologies (e.g., winnowers, gravity tables, screens), which separate smaller, less dense grains and aspirate out fines, can reduce mycotoxin levels in maize and wheat [36,37,38,39,40]. Similar principles can be applied in a more affordable and accessible manner, although there can be accompanying tradeoffs in efficiency and effectiveness. For example, a relatively inexpensive (and low-throughput) optical sorting system could reduce aflatoxins and fumonisins in maize from Kenyan markets based on visible and near infrared reflectance [41]. However, an affordable and high-throughput sorting device is not readily available for users in low-resource settings.

Sorting can also offer insights into the relationship between maize kernels and a fungal pathogen. Among corn screenings sieved into different classes, the smaller particles were highly associated with fumonisin contamination, suggesting that small or broken kernels were more susceptible to contamination [42]. Single kernel analysis of South African maize demonstrated that visually symptomatic kernels had significantly higher fumonisin levels [43]. Outside the sorting context, maize kernel bulk density (test weight) was negatively correlated with fumonisin in a maize mapping population [44,45]. Further insights into such relationships are necessary as they can help to guide and enhance sorting efforts.

To reduce fumonisin content and explore kernel characteristics associated with contamination, we developed a low-cost (materials approximately USD 300) sorting machine called the “DropSort” that employed established grain sorting principles. The DropSort used vacuum suction to aspirate fines and sort out small, light, and low-density kernels that were considered more likely to be toxic. This device has also been used on aflatoxin- or fumonisin-contaminated maize and groundnut samples, and it was most effective at sorting fumonisin-contaminated maize [46,47]. We expanded on this work by (1) using 3D-printed plastic kernels of varying masses and densities to validate and characterize this device and propose best practices for use, (2) assessing the DropSort’s effects on bulk fumonisin concentrations and associated bulk physical characteristics in unsorted maize and three sorted fractions, and (3) analyzing single kernel characteristics from two of the sorted fractions to determine their relationships with fumonisins.

## 2. Results

### 2.1. Plastic Kernel Sorting

**Figure 1:**
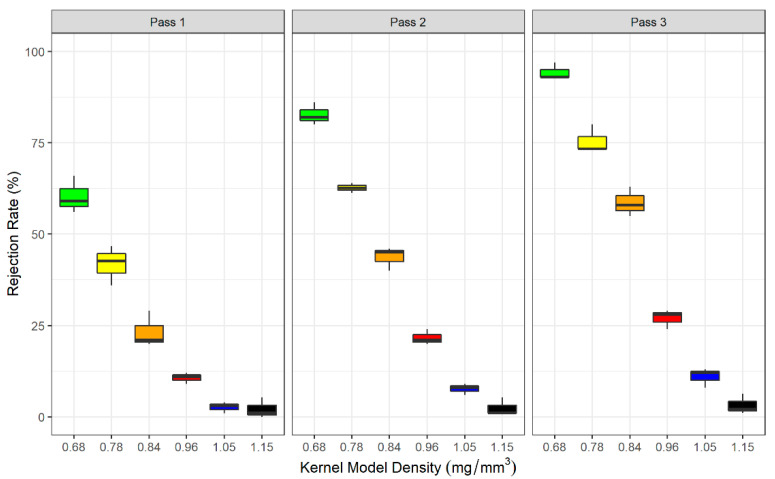
DropSort rejection rates (three trials) of six plastic kernel model sets at Setting 15 and across three passes of re-sorting the accepted fraction. Within each pass, kernel models’ mean rejection rates were significantly different (1-way ANOVAs: Pass 1 *p*-value = 9.40 × 10^−9^, Pass 2 *p*-value = 1.86 × 10^−12^, Pass 3 *p*-value = 1.16 × 10^−13^). Within each pass, post hoc pairwise *t*-tests were performed between each kernel model set, and different letters represent significant differences between kernel model groups (*p*-value < 0.05).

Six distinct plastic kernel model sets were created that had the same volume but different masses and densities, and rejection rates were measured at each setting for multiple re-sorting passes (Supplementary Table S1). The rejection rates of these kernel model sets varied significantly across passes and settings. For example, all models’ rejection rates were significantly different at Setting 15 except for the heaviest and densest three kernel models in Pass 1 (Figure 1). This demonstrated that the DropSort was able to separate plastic kernel models based on their mass and density.

**Figure 2:**
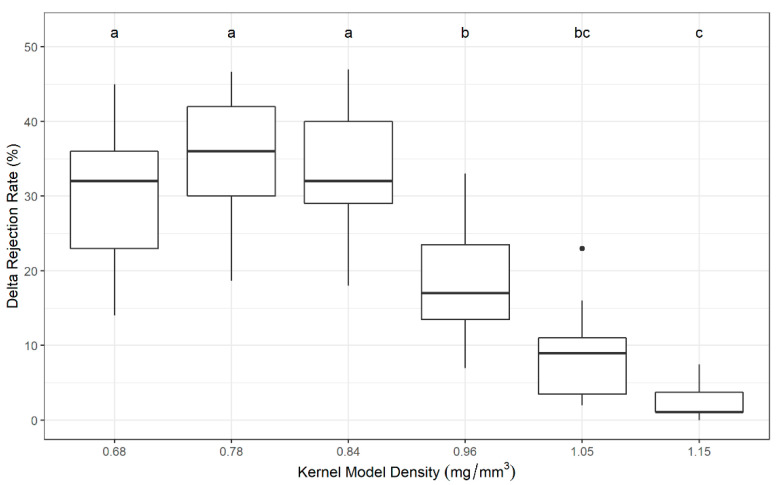
Differences in rejection rate at Pass 3 and Pass 1 (Delta Rejection Rate (DRR)) for each kernel model group. Group means differed significantly (1-way ANOVA, *p*-value = 3.7 × 10^−7^). Post hoc pairwise t-tests were performed between them, and different letters represent significant differences between kernel model groups; the three heaviest/densest kernel models had a lower DRR (*p*-value < 0.05).

The effect of using multiple passes to re-sort the accepted fraction was examined by analyzing the change in rejection rate from the first pass to the third pass (delta rejection rate or DRR = Pass 3 rejection rate − Pass 1 rejection rate). The DRR differed significantly across kernel models and was significantly higher for lighter/lower density kernel models (Figure 2). By re-sorting the accepted fraction over multiple passes, lighter and less dense kernel models were rejected more frequently than heavier and denser kernel models.

**Figure 3:**
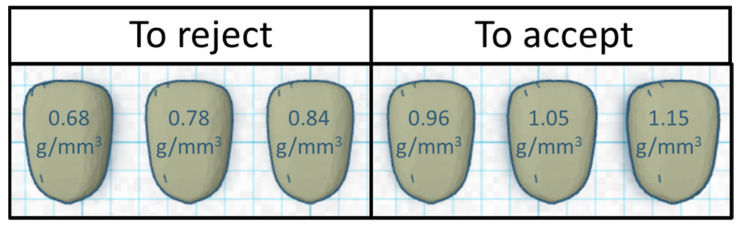
Cartoon depictions of the six plastic kernel model sets labeled with their respective densities. An arbitrary classification cutoff was applied to further explore sorting performance of the DropSort. The three heaviest/densest models (≥0.96 mg/mm^3^) were classified as the “to accept” class and the lightest/least dense three kernel models (≤0.84 mg/mm^3^) were classified as the “to reject” class.

Next, we compared the DropSort’s sorting performance to an arbitrarily designated accept/reject cutoff. Because the DRR was lower for the three denser and heavier kernel models, the three lightest/least dense kernel models were designated as the “to reject” (i.e., “toxic”) class and the heavier and denser kernel models as the “to accept” (i.e., “clean”) class (Figure 3).

**Figure 4:**
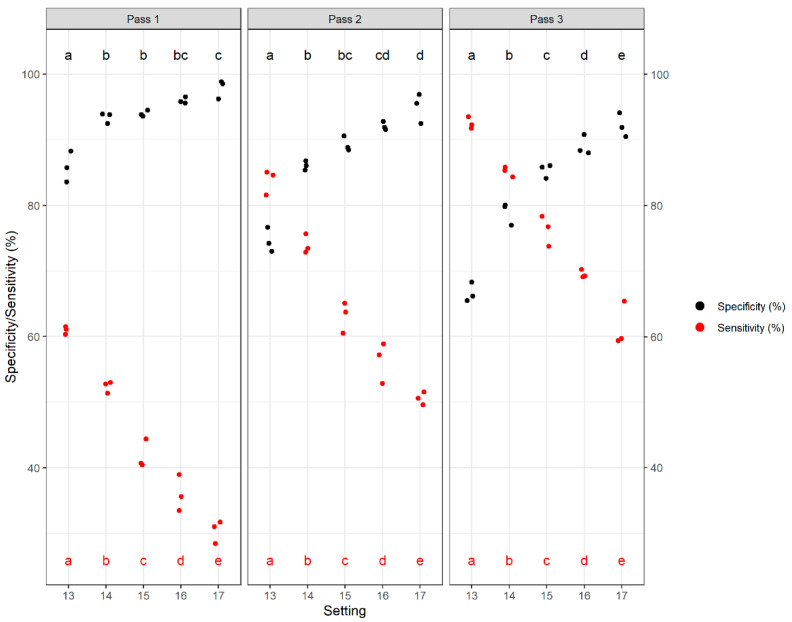
Specificity and sensitivity rates of the DropSort’s three trials of sorting plastic kernel models at different settings and over three passes. Within each pass, mean sensitivity and specificity rates varied significantly across settings (1-way ANOVAs: sensitivity Pass 1 *p*-value = 6.30 × 10^−11^, sensitivity Pass 2 *p*-value = 1.31 × 10^−10^, sensitivity Pass 3 *p*-value = 3.22 × 10^−12^, specificity Pass 1 *p*-value = 9.06 × 10^−6^, specificity Pass 2 *p*-value = 6.57 × 10^−7^, specificity Pass 3 *p*-value = 9.65 × 10^−8^). Post hoc pairwise t-tests were performed between each group, and different letters (red letters for sensitivity, black letters for specificity) represent significant pairwise differences between settings (*p*-value < 0.05).

Within this framework, we calculated how well the DropSort classified true positives (sensitivity = toxic rejected kernels/total rejected kernels) and true negatives (specificity = clean accepted kernels/total accepted kernels) across passes and settings. Sensitivity and specificity are important metrics for end users that reflect utility for food security (high sensitivity means few “clean” kernels are rejected) and food safety (high specificity means few “toxic” kernels are accepted), respectively. We found that within each pass, increasing the setting of the DropSort had a positive relationship with specificity and a negative relationship with sensitivity. The trends reversed across passes, with specificity decreasing and sensitivity increasing. The crossover points in Pass 2 and Pass 3 represent the setting with optimal sorting accuracy for that pass (Figure 4).

**Figure 5:**
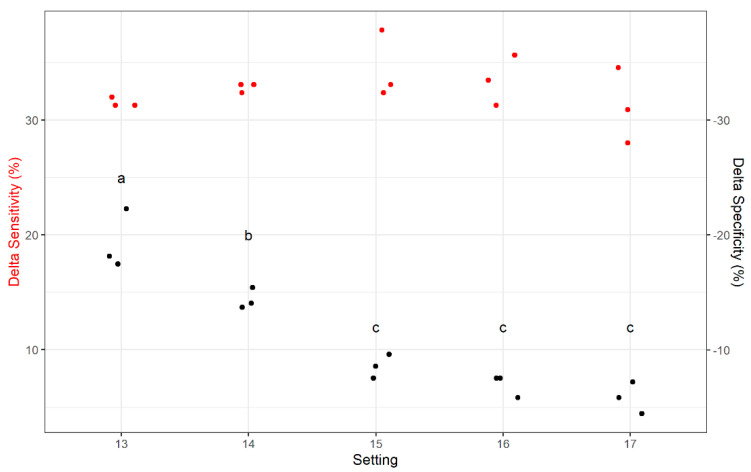
Differences in sensitivity (Delta Sensitivity) and specificity (Delta Specificity) between Pass 3 and Pass 1 at Settings 13–17. The three heaviest/densest classes of plastic kernel models were classified as “to accept” and the three lightest/least dense classes of plastic kernel models as “to reject”. Across settings, mean differences were significantly different for Delta Specificity (1-way ANOVA, *p*-value = 5.81 × 10^−7^), but not for Delta Sensitivity (1-way ANOVA, *p*-value = 0.98). This suggests that specificity can be improved without a loss of sensitivity by using multiple passes and a higher setting. Different black letters represent significant pairwise differences in Delta Specificity between settings (*p*-value < 0.05).

Although the positive relationship between number of passes and sensitivity indicated the DropSort’s performance was improving, it is necessary to consider the tradeoff in decreased specificity. To explore this, we defined Delta Specificity and Delta Sensitivity as the difference in specificity and sensitivity between Pass 3 and Pass 1. The gain in sensitivity from Pass 1 to Pass 3 was greater than the loss in specificity at all settings (Figure 5). Although there was no change in Delta Sensitivity across settings, the loss in specificity was less pronounced at the higher settings 15–17 (Figure 5).

### 2.2. Bulk Maize Sorting

The results of the plastic kernel sorting guided the methods used for sorting maize that was naturally infected by F. verticillioides and contaminated with fumonisins. Sorting each of the 24 samples of maize with the DropSort created a heavy fraction (HF), medium fraction (MF), and light fraction (LF). MF was included to add more insight into how much maize needs to be removed to reduce fumonisin levels with the DropSort (Supplementary Table S2). As a proportion (mean ± SE) of the original unsorted mass, HF was the largest (0.70 ± 0.11), followed by MF (0.21 ± 0.07), and LF (0.09 ± 0.05).

**Figure 6:**
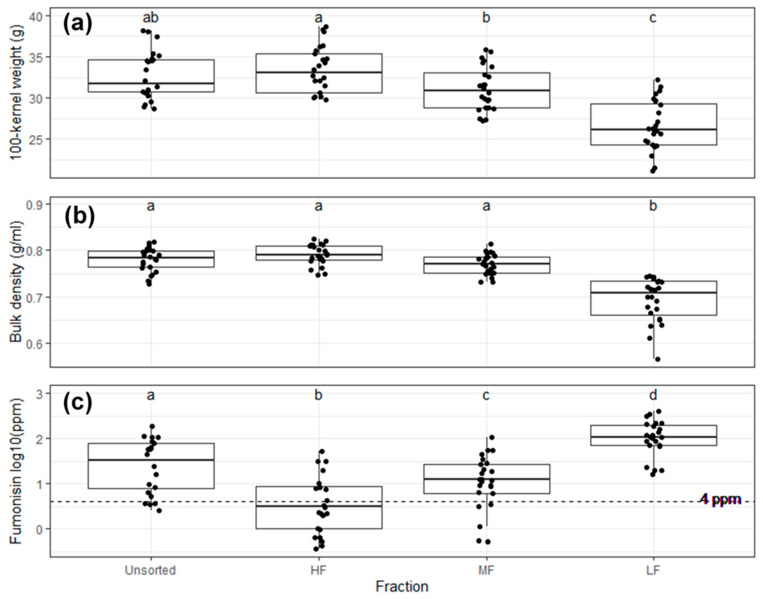
Mean bulk trait values of the unsorted, heavy fraction (HF), medium fraction (MF), and light fraction (LF). (**a**) Mean 100-kernel weight differed significantly among fractions (1-way ANOVA, *p*-value = 8.01 × 10^−13^). LF 100-kernel weight was significantly lower than all other fractions, and MF 100-kernel weight was significantly lower than HF (different letters represent pairwise t-test *p*-value < 0.05). (**b**) Bulk density varied significantly among fractions (1-way ANOVA, *p*-value = 2.20 × 10^−16^), and LF bulk density was significantly lower than all other fractions (different letters represent pairwise *t*-test *p*-value < 0.05). (**c**) Log10-transformed fumonisin differed significantly among fractions (1-way ANOVA, *p*-value = 4.63 × 10^−13^). Dashed line indicates 4 ppm regulatory limit. Compared to unsorted, HF was significantly lower, MF no difference, and LF significantly higher (different letters represent pairwise t-test *p*-value < 0.05).

Sorted fractions had significantly different bulk characteristics. The 100-kernel weight and bulk density were significantly lower in LF compared to all other fractions, and for the 100-kernel weight, MF was significantly lower than HF (Figure 6A,B). Compared to the unsorted samples, log_10_-transformed fumonisin was significantly reduced in HF, did not differ in the MF, and was significantly higher in the LF (Figure 6C). Relative to the original unsorted samples, the percent differences in fumonisin (mean and median) for each fraction were HF (−15.39% and −89.06%), MF (64.72% and −23.74%), and LF (652.28% and 230.83%). The DropSort effectively reduced fumonisin in HF relative to the bulk sample by concentrating the toxin in LF. In relation to the 4 ppm FDA guidance level, safe samples were most frequent in HF (14/24) followed by MF (5/24), unsorted (4/24), and LF (0/24).

We also explored whether any of the bulk characteristics were correlated with bulk fumonisin in the unsorted samples. Spearman rank correlations between the bulk characteristics of each fraction and log_10_-transformed fumonisin values yielded a single significant correlation: LF 100-kernel weight was negatively correlated with unsorted log_10_-transformed fumonisin (Table 1). This correlation could be an inexpensive proxy for unsorted fumonisin.

### 2.3. Single Kernel Analysis

**Figure 7:**
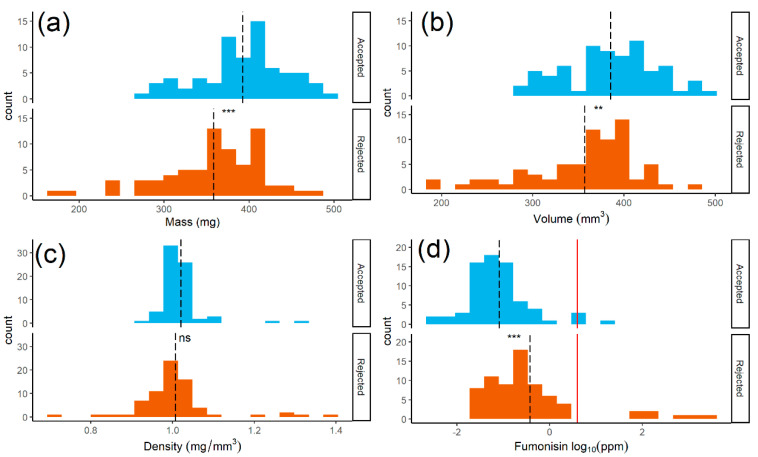
Mass, volume, density and log_10_-transformed fumonisin distributions for 72 individual maize kernels from the “accepted” heavy fraction (HF; light blue) and 72 kernels from the “rejected” light fraction (LF; orange). Dashed lines indicate group means, and asterisks represent a significant difference between accepted and rejected kernels. (**a**) Mean HF kernel mass was significantly higher than that of LF kernels (two-sided unpaired *t*-test, *p*-value = 2.9 × 10^−4^). (**b**) Mean HF kernel volume was significantly higher than that of LF kernels (two-sided unpaired *t*-test, *p*-value = 2.2 × 10^−3^). (**c**) There was no significant difference in mean density between HF and LF kernels (two-sided unpaired *t*-test, *p*-value = 0.33). (**d**) Mean log10-transformed fumonisin was significantly greater in LF kernels compared to HF kernels (two-sided unpaired *t*-test, *p*-value = 2.4 × 10^−5^), and solid red line indicates 4 ppm regulatory limit.

For a single representative bulk sample, 72 kernels each were selected at random from HF and LF to examine their individual characteristics (Supplementary Table S3). Compared to its unsorted fraction, this sample had lower fumonisin in HF and higher levels in MF and LF (Table 2). The single kernel mean volume and mass was lower in LF compared to HF, but density was not significantly different among fractions (Figure 7A–C). Notably, the density distributions were narrower than those for mass and volume. The DropSort reduced mean fumonisin concentrations in the HF relative to the bulk sample, and high fumonisin outliers were found in the LF (Figure 7D). At a 4 ppm cutoff, only 9/144 kernels were found to exceed this limit, but more of these toxic kernels were found in LF (*n* = 7) compared to HF (*n* = 2).

**Figure 8:**
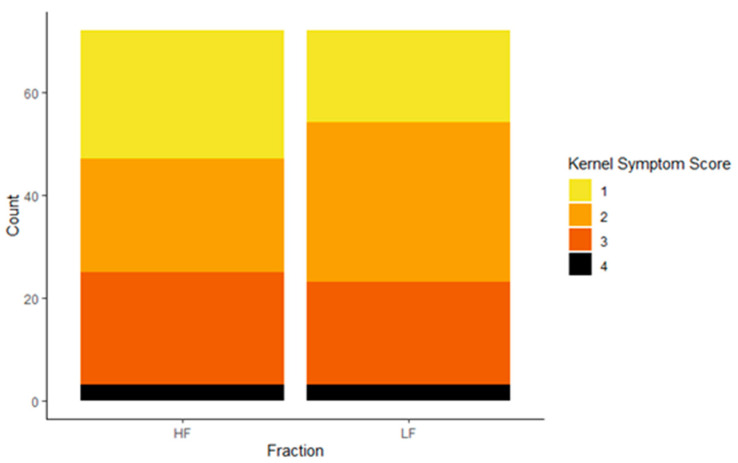
Counts of different kernel symptom types in heavy fraction (HF; *n* = 72) and light fraction (LF; *n* = 72). Fusarium kernel rot symptom scoring was modified from Morales et al. (2019), and kernels were scored as 1 = asymptomatic, 2 = starburst, 3 = purple, and 4 = mummified. The proportions of symptom types did not differ between HF and LF (chi-squared test, *p*-value = 0.90).

Symptoms were scored on all single kernels using four categories. The proportions of kernels in each category did not differ between the two fractions, indicating that the DropSort did not affect kernel appearance (Figure 8).

**Figure 9:**
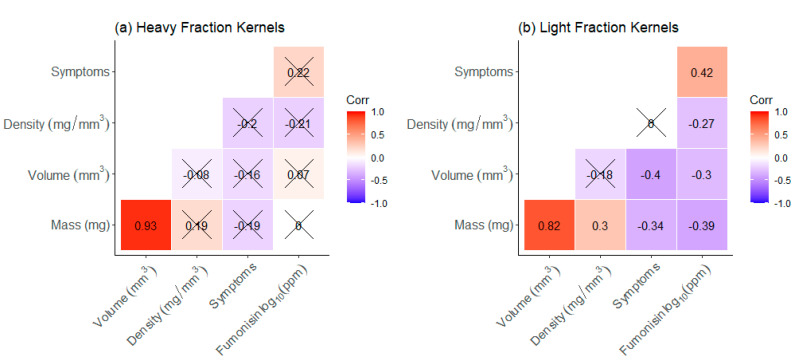
Spearman rank correlations among five single kernel traits (mass, volume, density, visually scored symptoms, and log_10_-transformed fumonisin) for 72 heavy fraction kernels (**a**) and 72 light fraction kernels (**b**) (overlaid X indicates *p*-value ≥ 0.05).

To understand how different kernel characteristics were related, Spearman rank correlations were calculated within HF and LF subsets. Examining the correlations for HF kernels was uninformative, as there were no significant correlations except for the expected strong positive relationship between mass and volume (Figure 9a). However, LF kernels revealed numerous significant correlations where the three physical kernel attributes (mass, volume, and density) were negatively correlated with log_10_-transformed fumonisin (Figure 9b). Moreover, despite not being affected by the DropSort, symptom type was positively correlated with fumonisin in LF (Figure 9b). The relationships among these five kernel traits were distinctly stronger among the LF kernels than among HF kernels.

## 3. Discussion

The DropSort was designed to be a low-cost and high-throughput option for reducing the mycotoxin load in a sample of maize. The intention was to leverage the hypothesized negative correlation between kernel mass/volume/density and fumonisin concentration. To be a feasible option for fumonisin mitigation (especially in low-resource settings), considerations of both food safety (fumonisin reduction) and food security (amount of grain removed) had to be taken into account. We showed that the DropSort reduced bulk fumonisins, and that it could be used to generate an inexpensive proxy for bulk fumonisin.

Using plastic kernel models, we demonstrated that the DropSort rejected kernel models of different masses/densities at variable rates. This means that, for a given a sample of grain, the rejection rate could be adjusted by the choice of setting (the position of the bar that cut the flow of grain between the two chambers) and number of passes (re-sorting the accepted/heavy fraction).

Analyzing plastic kernel models also offered insights into the crucial tradeoff between sorting sensitivity and specificity. Sensitivity is the rate of true positives (toxic kernels), which has implications for food security, as high sensitivity means that fewer clean kernels are rejected and discarded. In contrast, the rate of true negatives (clean kernels) in specificity has implications for food safety, as it reflects the toxicity of accepted kernels (i.e., low specificity means that many “toxic” kernels will be accepted). Balancing specificity and sensitivity is a challenge, and as expected, there was a clear tradeoff between these two metrics using an arbitrary mass/density cutoff. Encouragingly, altering certain parameters optimized these tradeoffs. For instance, re-sorting the accepted fraction led to an increase in sensitivity, while the reduction in specificity was mitigated. Moreover, using a more selective setting (taking a smaller cut of the grain with each pass through the sorter) allowed specificity to be improved through multiple passes without affecting sensitivity. These results suggest sensitivity/specificity tradeoffs can be optimized by being more selective at each pass and re-sorting the accepted fraction over multiple passes. Although this approach clearly improves sorting performance, re-sorting the accepted fraction increases the amount of time needed to sort. It would, in principle, be possible to modify the design of the device to enable continuous re-sorting at a very conservative setting.

The results of plastic kernel model sorting provided guidance on how to sort real maize grain. We found that, compared to bulk density, 100-kernel weight was more sensitive to sorting. This suggested that the DropSort was more effective at stratifying the sample based on kernel mass rather than on density.

The primary purpose of the DropSort was to reduce mycotoxin concentrations in bulk maize. It successfully reduced fumonisin in the heavy fraction compared to unsorted maize by concentrating contaminated kernels mainly in the light fraction and, to a lesser extent, the medium fraction. However, it was not fully effective at reducing fumonisin concentration below the *Codex Alimentarius* standard and FDA guidance level of 4 ppm. In HF, more than half of the samples were below the 4 ppm regulatory limit, while none of the light fraction samples were below the regulatory limit. These fumonisin reduction results were similar to previous findings with the DropSort that had single “accept” and “reject” fractions (Aoun et al. 2020, Ngure et al. 2021), and additional insight was gleaned from observing the level of fumonisin in the medium fraction as being an intermediate between the heavy and light fractions. To reduce fumonisins in maize more effectively, the DropSort should be integrated with complementary methods such as screening, washing, flotation, spectral sorting, and/or visual/hand sorting [24,28,30,41]. In fact, by combining the DropSort with size screening, maize fumonisin concentrations were reduced well below regulatory limits [46].

Analysis of single kernels from the heavy and light fractions provided insights on how the DropSort affected physical kernel attributes, as well as how those attributes relate to fumonisin contamination. To our knowledge, this is the first study to analyze both the fumonisin content and physical characteristics of single kernels. As implicated by 100-kernel weight and bulk density results, the mass and volume of light-fraction kernels were significantly lower than heavy-fraction kernels. Surprisingly, density was not affected. Density had a much narrower distribution than mass and volume, and density did not differ between kernels of the heavy and light fractions. This suggested that density, despite its negative correlation with fumonisin, did not have sufficient variation to allow effective density-based sorting.

Correlations among physical features (mass, volume, and density) and fumonisin in the light fraction yielded modest negative correlations, showing that light/small/low-density kernels were more likely to have high fumonisin concentrations. These significant negative correlations with fumonisin corroborate previous research on the relationship between fumonisin and maize bulk characteristics such as 100-kernel weight or screenings [42,46,47]. It is not clear whether these correlations occur because smaller, lighter, and/or less dense kernels are more vulnerable to infection, because fungal pathogen colonization reduces kernel mass and density, or because fungal infection retards/arrests kernel growth and development. Regardless, these results provide insights on the opportunities and limitations of sorting fumonisin-contaminated maize.

Past studies have demonstrated the effectiveness of visual sorting on bulk samples, and single-kernel analysis previously documented that unhealthy kernels have high fumonisin concentrations compared to healthy kernels [26,28,29,43]. We showed that discolored and damaged kernels were positively associated with fumonisin, but only in the light fraction. This suggests that kernels can be discolored for a variety of reasons, only some of which may involve a given mycotoxigenic fungus.

Although initially designed as a scaled down technology for mycotoxin mitigation in low-resource contexts, there are other potential uses for the DropSort. There are incentives for multiple stakeholders to generate rapid and inexpensive proxies for fumonisin contamination. For example, a trader may want to quickly assess whether a sample is likely to be rejected, or a plant breeder would be interested in a cheaper alternative to expensive mycotoxin assays in disease trials. Previous studies found that kernel bulk density (test weight) was negatively correlated with Fusarium ear rot and fumonisin concentration [44,45]. These results were found for inoculated grain, whereas the current study utilized naturally infected grain. Such relationships were expected be less obvious under non-inoculated conditions, so the ability of the DropSort to identify a significant proxy was encouraging. The significant negative correlation between fumonisin and LF 100-kernel weight suggests that this measure could function as a predictor of fumonisin contamination. Although this proxy (ρ = −0.51) would not replace validated fumonisin assays, it could still be beneficial in certain situations. Measuring LF 100-kernel weight to screen for high and/or low fumonisin concentrations could reduce costs in many scenarios and expand screening of putatively contaminated grain.

## 4. Conclusions

Effectively sorting mycotoxin-contaminated maize is a significant challenge as tradeoffs of cost, food safety, food security, and time must be considered. Consumers, producers, and traders have incentives to sort mycotoxin-contaminated maize, but options to do so are limited in low-resource contexts. Presently, visual sorting is a widely adopted method, and it has been shown to be moderately effective for reducing fumonisin levels. A mechanical sorting device could offer a more time-efficient alternative and/or could complement existing methods. The DropSort could prove valuable in resource-limited areas in which maize is the staple crop, fumonisin contamination is common, and maize is milled in small batches. It could be integrated within existing local infrastructure in the grain processing chain such as local grain mills, small-medium scale millers, or traders. Addressing the persistent and pervasive challenge of fumonisin contamination in human food requires solutions that are adaptable, suited to a community’s needs and resources, and part of a multifaceted approach. The capacity to mitigate food safety challenges should be universal, and in the context of fumonisin contamination, devices like the DropSort can be part of an effective strategy.

## 5. Materials and Methods

### 5.1. DropSort Device

The DropSort device was designed by The Widget Factory (Ithaca, NY) and functioned by applying suction to free-falling kernels, in a manner conceptually similar to winnowing. A Grizzly G0710 1 hp wall hanging dust collector (blower) was used to generate negative pressure; the blower has a flow rate of 537 feet^3^/minute (Grizzly Industrial^®^, Bellingham, WA). The blower was connected to the DropSort with a 10 cm diameter tube attached to the back of the sorting space. The three-dimensional sorting space (length = 104 cm, width = 58 cm, depth = 2.5 cm) had an adjustable dividing arm at its base that separated two removable bins. A hopper was located on top of the device, and a mechanical feeder shook individual kernels into the sorting space. Each kernel dropped from the top left of the device and, as it fell, was subjected to negative air pressure that caused the kernel to move from left to right towards the fan tube attachment. The blower generated constant suction, and a kernel’s path was determined by the interaction of its physical properties with negative pressure in the sorting space. The dividing arm could be adjusted from Setting 1 to Setting 20 (left to right; 60° to 120°) to change the proportion of kernels accepted or rejected. The left bin disproportionately collected larger, heavier, and/or denser kernels and was the putative “accept” bin, while smaller, lighter, and/or less dense kernels were more likely to accumulate in the putative “reject” bin (right). The total cost of materials was approximately USD 300, the majority of which was the cost of the blower.

### 5.2. Plastic Kernel Printing

Plastic kernel models were generated in the webapp TinkerCAD and converted to 3D-printable gcode files in Cura version 15.04.2 (Ultimaker, Utrecht, Netherlands). In Cura, G-Code files were generated from STL files with six different infill densities (100%, 80%, 60%, 40%, 30%, and 20%) to create plastic kernels of identical volume but different masses and densities (Supplementary File S1). The expected volumes of the plastic kernel models were calculated in Rhino3D; the masses were measured on a scale, and density was calculated as mass/volume (Robert McNeel & Associates, Seattle, WA, USA).

Sets (*n* = 100) of each plastic kernel model were printed on a Kossel Model A 3D Printer with 1.75 mm diameter polylactic acid (PLA) filament at 210 °C (Turn Key Innovations, Ashby, MA, USA). To easily distinguish between different kernel models, different colored PLA was used for each infill density.

### 5.3. Plastic Kernel Sorting

The DropSort device was used to sort all plastic kernel models at settings 13, 14, 15, 16, and 17, and then, the accepted fraction was re-sorted twice for a total of three passes through the sorter. After each pass, the number of each plastic kernel type in each bin was recorded. This procedure was repeated three times at each setting.

Sorting performance was assessed by calculating the rejection rate of the various kernel models at each sorting and pass combination. To calculate the sensitivity and specificity, an artificial arbitrary cutoff was created in which the heaviest/densest three kernel models (≥0.96 mg/mm^3^) were classified as the accepted class and the lightest/least dense three kernel models (≤0.84 mg/mm^3^) were the rejected class. Using this cutoff, the possible outcomes for each kernel model were true positive (A), false positive (B), false negative (C), or true negative (D). The rate of true positives, sensitivity, was defined as A/(A+C), and the rate of true negatives, specificity, was D/(B+D). To demonstrate how multiple passes affected these three sorting parameters at each setting, the difference between the third pass value and the first pass value was calculated.

### 5.4. Texas Maize

A total of 24 samples of shelled yellow dent maize were acquired from north Texas. These samples were naturally infected with *Fusarium verticillioides* and displayed symptoms (e.g., starburst kernels) of Fusarium kernel rot. To DropSort these samples, 1 kg from each sample was passed through the DropSort, and the accepted fraction was sorted an additional two passes at setting 14. After the initial three passes, the kernels in the rejected bin were removed and stored as the light fraction (LF). The remaining accepted fraction was sorted for an additional three passes at setting 12. The kernels in the rejected bin were designated as the medium fraction (MF), and the remaining kernels in the accepted bin were designated as the heavy fraction (HF). The exact combination of settings and passes had been selected by testing initial samples to find a setting that would sort approximately 10% of the original mass into LF, 20% into MF, and 70% into HF.

The following were measured on each unsorted sample and their associated HF, MF and LF subsamples:

Mass: The total mass (g) of each fraction was measured on a PG403-S DeltaRange^®^ scale (Mettler Toledo, Columbus, OH, USA).

Bulk Density: A 200 mL sample of maize was randomly taken from its fraction, measured in a 250 mL graduated cylinder, and weighed. This was replicated three times for each fraction. If there was less than 200 mL of maize in the sample, the volume was recorded and only measured once. Bulk density was calculated by dividing the mass of each sample by its volume.

100-kernel Weight: For each fraction, 100 kernels were randomly selected and weighed. This procedure was replicated three times. In cases where the number of kernels in a fraction did not exceed 100, the total number of kernels and mass were recorded and converted to a 100-kernel weight.

Total fumonisins: A random 15 g sample (or entire sample if less than 15 g) was taken from each fraction and ground into a fine powder in an IKA Tube Mill (IKA^®^ Works, Inc, Wilmington, NC, USA). Fumonisins were extracted at a 1:2 (weight:volume) ratio in 90% methanol, vortexed for five minutes, and filtered through Whatman #1 filter paper. Sample extracts were then diluted 1:20 in distilled water. Fumonisin concentrations were quantified according to the Fumonisin ELISA Assay procedure (Helica Biosystems Inc., Santa Ana, CA, USA). Samples, standards, and a check were assayed in duplicate on each plate. The optical density of each well at 450 nm was read by a BioTek Synergy 2 multi-mode plate reader using Gen5 software (BioTek Instruments, Inc., Winooski, VT, USA). Sample concentrations were determined by fitting a standard curve to the standards’ OD values. As a check, a validated 3.5 µg/g (ppm) sample of fumonisin-contaminated maize was tested on each plate. To ensure data quality, and coefficient of variation (CV) cutoffs were 10% for replicate samples within a plate and 15% for the between plate check. Samples were re-assayed if they exceeded these CV cutoffs. Samples that exceeded the 6 ppm maximum concentration were diluted in distilled water and re-assayed again on a new ELISA plate.

### 5.5. Single Kernel Analysis

One sample was analyzed further at the single kernel level. A total of 144 kernels were randomly selected: 72 from the heavy fraction and 72 from the light fraction.

The mass of each kernel was measured on a scale (Intelligent Laboratory Classic Top Loading Balance, 100 g × 0.001 g). Each kernel’s volume was calculated using a modified version of a phenoSeeder that we created (Jahnke et al. 2016). The device consisted of a stepper motor, a Raspberry Pi 3 Model B (Raspberry Pi Foundation, Cambridge, UK), and an Arducam M12 Lens Camera (Arducam, Nanjing, China). Briefly, a kernel was placed crown-side down on a pedestal powered by the stepper motor that rotated the kernel in one full 360° rotation, pausing at 10° increments. At each pause, a photo was taken for a total of 36 images per kernel. Camera parameters were adjusted in the python code from Jahnke et al. (2016). This code cropped, masked, and stitched each image together to create a 3D reconstruction of the kernel, and the volume of each kernel was calculated from this 3D reconstruction.

Each kernel was scored for Fusarium kernel rot on a 1–4 scale: 1 = asymptomatic, 2 = starburst, 3 = purple, 4 = mummified (adapted from Morales et al. 2019). This scale differs from Morales et al. (2019) in excluding the “blush” kernel category, as this kernel type was not observed in our material.

Each kernel was ground by a ball bearing in SPEX Geno/Grinder 2000 Sample Prep Shaker Tissue Homogenizer (New Life Scientific, Inc., Cridersville, OH, USA). Fumonisins were extracted at a 1:2 (mass to volume) ratio in 90% methanol in 2.0 mL Eppendorf tubes, vortexed for five minutes, and centrifuged at 14,000 rpm for 10 min. The supernatant was removed and placed in a 1.5 mL Eppendorf tube; then, fumonisins were quantified from diluted supernatant according to the protocol described above. The same check and CV cutoffs were used, and any samples exceeding 6 ppm were diluted in distilled water and re-assayed.

### 5.6. Data Analysis

All data analyses and statistics were performed in R 4.03, and the ggplot2 and ggcorrplot packages were used for visualizations [48,49,50]. To compare among group means for plastic kernels and bulk maize, one-way ANOVAs were performed, and if significant, pairwise t-tests with a Holm *p*-value adjustment were used to compare between group means. Unpaired two-sided Student’s t-tests were used for single kernel characteristic means. For assessing bulk and single kernel trait relationships, Spearman rank correlations were calculated. Fumonisin concentrations were log_10_-transformed for statistical analyses because the data were highly skewed and not normally distributed according to the Shapiro–Wilk normality test.

## Figures and Tables

**Table 1:** Spearman rank correlations between fumonisin concentrations and bulk characteristics in the unsorted samples, the heavy fraction (HF), medium fraction (MF), and the light fraction (LF) of each fraction (ns *p*-value > 0.05; * *p*-value = 0.012).

Spearman Correlations	Unsorted 100-Kernel Weight	Unsorted Bulk Density	HF 100-Kernel Weight	HF Bulk Density	MF 100-Kernel Weight	MF Bulk Density	LF 100-Kernel Weight	LF Bulk Density
Unsorted Fumonisin log_10_(ppm)	−0.24 ns	−0.08 ns	−0.34 ns	−0.12 ns	−0.24 ns	−0.13 ns	−0.51 *	−0.22 ns

**Table 2:** Bulk characteristics of the unsorted, heavy fraction (HF), medium fraction (MF), and light fraction (LF) for the sample used in single kernel analysis.

	Mean 100-Kernel Weight (g)	Mean Bulk Density (g/mL)	Fumonisin (ppm)
Unsorted	37.38	0.77	23.47
HF	38.60	0.78	0.65
MF	34.88	0.77	55.20
LF	25.64	0.65	305.84

## Data Availability

All analyzed data are available in this article’s Supplementary Tables.

## Supplementary Materials

The following are available online at https://www.mdpi.com/article/10.3390/toxins13090652/s1. Table S1: Rejection rates of each plastic kernel model set at all combinations of settings and passes. Table S2: Bulk characteristics of the unsorted maize samples and the three sorted fractions. Table S3: Single kernel characteristics of 72 heavy fraction and 72 light fraction maize kernels. File S1: STL file for the plastic kernel models.
